# Quantifying the Occurrence of High-Risk Pregnancy: A Comprehensive Survey

**DOI:** 10.7759/cureus.59040

**Published:** 2024-04-26

**Authors:** Alby Johnson, Sasi Vaithilingan, Latha Ragunathan

**Affiliations:** 1 Obstetrics and Gynaecology, Vinayaka Mission's Research Foundation, Salem, IND; 2 Child Health Nursing, Vinayaka Mission's College of Nursing, Puducherry, Vinayaka Mission's Research Foundation, Puducherry, IND; 3 Microbiology, Aarupadai Veedu Medical College and Hospital, Vinayaka Mission's Research Foundation, Puducherry, IND

**Keywords:** occurrence of high-risk pregnancy, pregnancy risk factors, disorders in pregnancy, pregnancy complications, pregnancy-induced hypertension, hypothyroidism in pregnancy, high-risk pregnancy

## Abstract

Background

High-risk pregnancies are characterized by various factors that pose potential risks to maternal and newborn health outcomes. Early detection of these high-risk pregnancies serves as a crucial initial step in preventing maternal mortality and morbidity, thereby promoting the overall health of both mother and baby. This study sought to assess the occurrence of high-risk pregnancy and investigate the factors associated with it among pregnant women.

Methods

A descriptive survey was undertaken at the Obstetrics and Gynaecology outpatient department of a District Government Hospital in Tamil Nadu, involving 1889 pregnant women in their second and third trimesters. A structured questionnaire, constructed following the Indian standard criteria outlined by the National Health Portal of India, served as the data collection tool. The survey was conducted in February and March 2022, during which pregnant women were interviewed. Subsequently, the collected data underwent descriptive and inferential statistical analysis.

Results

Among the 1889 pregnant women surveyed, 29% (n=530) were classified as high-risk pregnancies. Within this group, 34.3% (n=182) were diagnosed with hypothyroidism, while 23.2% (n=123) experienced pregnancy-induced hypertension. Significant associations with high-risk pregnancy were observed for factors such as age, education status, occupation, family income, socioeconomic status, and gravida among the pregnant women.

Conclusion

Policymakers must urgently implement evidence-based interventions aimed at early detection and treatment of high-risk pregnancies. This proactive approach is essential in preventing maternal mortality and morbidity.

## Introduction

The term "high risk" can evoke uncertainty for pregnant women, given its implications for both the mother and the newborn [1]. This complexity arises from various factors that may adversely affect either the mother, the newborn, or both. Globally, approximately 20 million pregnancies are classified as high-risk, leading to around 800 maternal deaths due to pregnancy-related complications [2]. In India, nearly 20-30% of pregnancies fall into this category, contributing to 70% of maternal mortality and morbidity [3]. Even pregnancies not categorized as high risk can still encounter complications due to the presence of one or more risk factors, such as age being over 35 or under 17, lifestyle choices, pregnancy-induced health issues, and pre-existing medical conditions. These factors can exacerbate the challenges associated with pregnancy [4].

Preventing complications during pregnancy hinges on vigilant monitoring, skilled care, and timely obstetrical interventions for expecting mothers [5]. Global maternal health initiatives concentrate primarily on antenatal, intranatal, and postnatal care to alleviate the burden of maternal mortality and morbidity. According to global data, approximately 87% of women have access to healthcare facilities at least once during their pregnancy [6]. Antenatal care (ANC) visits are essential for ensuring a safe pregnancy through regular health assessments, specialized pregnancy care, and screening for high-risk pregnancies (HRPs). The World Health Organization recommends a minimum of four to eight ANC visits, while in India, a minimum of four ANC visits is mandated [3]. Early identification of HRPs has the potential to reduce complications significantly and lower mortality and morbidity rates. Hence, all pregnant mothers need to undergo screening for high risk and those identified with high risk are to be followed up regularly. Studies on the prevalence of HRP in India are available; however, very few studies have been conducted to identify the magnitude of HRP in Southern parts of India. Thus, this study aims to assess the occurrence of HRPs and associated factors among pregnant women in this specific geographical area of Tamil Nadu.

## Materials and methods

The hospital-based descriptive survey was conducted in February and March 2022 at the Obstetrics and Gynaecology outpatient department, District Government Hospital, in Tamil Nadu. The study was approved by the Institutional Research Committee (VMCON-IRC-2021-2022-082). A consecutive sampling technique was adopted in this study. A total of 1889 pregnant women in the second and third trimesters who visited the antenatal clinic for regular antenatal check-ups were surveyed. The study included antenatal mothers who were proficient either in English or Tamil for communication, while those with psychiatric illnesses were excluded. A structured questionnaire was formulated under the Indian Standard Criteria outlined by the National Health Portal of India [3]. This questionnaire encompasses three parts. Part A dealt with the demographic characteristics of antenatal mothers such as age, educational status, occupation, family income, and religion of the participants. A modified Kuppuswami scale was used to identify the socioeconomic status [7]. Part B included the details regarding the obstetrical history of antenatal mothers like gravida of the pregnant mother, trimester of pregnancy, previous abortion history, previous cesarean section, bad obstetrical history, and present HRP status. Part C of the questionnaire was regarding the clinical parameters of antenatal mothers which included height, weight, body mass index, hemoglobin, blood group and type, blood glucose levels, thyroid hormone levels and blood pressure. The questionnaire was validated by the experts and reliability was determined (0.91).

The participants were recruited based on the inclusion criteria and the purpose of the study was explained to all study participants using participant information sheets, and written consent was obtained. Further, antenatal mothers were assured about the confidentiality of their information. The researcher conducted individual interviews with each participant and gathered the information along with their obstetric history. The height and weight of the participants were measured using a stadiometer and an adult weighing scale respectively. The body mass index (BMI) was calculated using the standard formula (weight in kilograms divided by height in meters squared). The hemoglobin level, blood group and type, blood glucose levels, and thyroid hormone levels were obtained from the participant's medical reports. The blood pressure was measured using a sphygmomanometer following standard procedures. Three consecutive readings were taken, each with a five-minute interval, and the average of these readings were recorded. The data were analyzed using Statistical Package for the Social Sciences (IBM SPSS Statistics for Windows, IBM Corp., Version 28.0, Armonk, NY). Descriptive statistics such as frequency, percentage, mean, and standard deviation were used to describe the data. The chi-square test was used to find the associated factors. The significance level was set at 0.05 for all the statistical analyses.

## Results

A total of 1889 pregnant women were enrolled in the present study. The mean age of participants was 25.01 ± 3.99. Most of the participants had diploma/intermediate education and were housewives (Table 1). The majority of the pregnant women were multigravida (52.9%) and were in the second trimester of pregnancy (28.7%).

**Table 1:** Distribution of demographic variables of antenatal mothers

Variable (n=1889)	Category	Frequency (f)	Percentage (%)
Age	<19	131	6.9
	20-24	782	41.4
	25-29	709	37.5
	30-34	234	12.4
	>35	33	1.7
Education	Primary school	93	4.9
	Middle school	290	15.4
	High school	330	17.5
	Intermediate/diploma	825	43.7
	Graduate/postgraduate	336	17.8
	Profession or honour	15	0.8
Occupation	Housewife	1330	70.4
	Unskilled worker	95	5
	Semi-skilled worker	114	6
	Skilled worker	133	7
	Clerical	35	1.9
	Semi profession	109	5.8
	Profession	73	3.9
Income	<3907	2	0.1
	3908-11707	649	34.4
	11708-19515	738	39.1
	19516-29199	416	22
	29200-39032	65	3.4
	39033-78062	19	1
Socioeconomic status	Lower	2	0.1
	Upper lower	1329	70.4
	Lower middle	379	20.1
	Upper middle	179	9.5
Religion	Hindu	1574	83.3
	Muslim	236	12.5
	Christian	79	4.2

**Figure 1:**
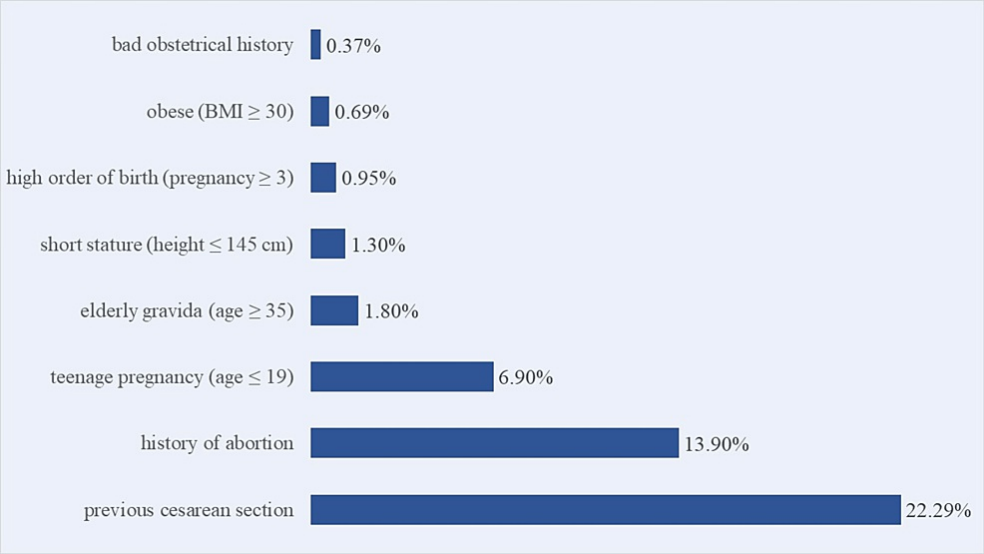
Risk factors in pregnancy

The prevalence of various risk factors among the antenatal mothers was identified. The most prevalent risk factor observed was a previous cesarean section, with 22.29% of the participants having undergone this procedure. The other risk factors were history of abortion (13.90%), teenage pregnancy (6.90%), elderly gravida (1.80%), short stature (1.30%), high order of birth (0.95%), obesity (0.69%), and bad obstetrical history (0.37%). These findings emphasize the importance of considering obstetric history and other demographic factors in maternal health assessments (Figure 1).

**Figure 2:**
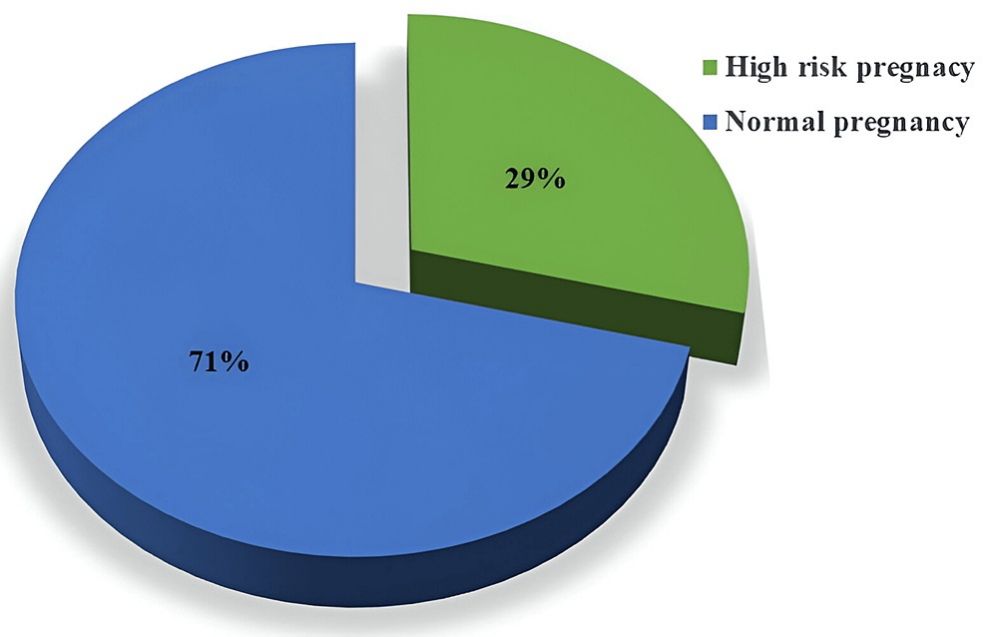
Distribution of high-risk pregnancy and normal pregnancy

While observing the distribution of HRPs versus normal pregnancies within the study cohort the data revealed that approximately one-third of the pregnant women fell into the category of HRPs (Figure 2).

Among the HRPs, hypothyroidism was the most prevalent high-risk condition, affecting 182 pregnant women (9.6%), followed by pregnancy-induced hypertension (6.5%), severe anemia (3.2%), and Rh incompatibility (2.8%). Other notable high-risk conditions included gestational diabetes mellitus (GDM) (2.5%), twin pregnancy (0.7%), oligohydramnios (0.6%), and hyperthyroidism (0.5%). Less common high-risk conditions included polyhydramnios, placenta previa, HBsAg positivity, seizure disorder, intrauterine growth retardation, cervical incompetence, HIV positivity, ovarian cyst, cholelithiasis, chronic hypertension, polycystic ovarian syndrome, and poliomyelitis, each affecting a small percentage of HRPs (Table 2).

**Table 2:** Distribution of high-risk conditions in pregnancy

High-risk conditions (n=1889)	Frequency (f)	Percentage (%)
Hypothyroidism	182	9.6
Pregnancy-Induced Hypertension	123	6.5
Severe Anaemia	60	3.2
Rh Incompatibility	53	2.8
Gestational Diabetes Mellitus	47	2.5
Twin Pregnancy	14	0.7
Oligohydramnios	11	0.6
Hyperthyroidism	10	0.5
Polyhydramnios	5	0.3
Placenta Previa	5	0.3
HBsAg Positive	5	0.3
Seizure Disorder	3	0.2
Intra Uterine Growth Retardation	3	0.2
Cervical Incompetence	2	0.1
HIV Positive	2	0.1
Ovarian Cyst	1	0.1
Cholelithiasis	1	0.1
Chronic Hypertension	1	0.1
Polycystic Ovarian Syndrome	1	0.1
Poliomyelitis	1	0.1
Normal	1000	71

The association of HRPs with various demographic variables showed that women aged 30-34 and over 35 experienced HRPs compared to younger age groups (p<0.001). Similarly, lower education levels, lower income, and lower socioeconomic status were associated with a higher incidence of HRPs (p=0.002, p=0.004, p<0.001, respectively). Moreover, a statistically significant association was found between the occupation of pregnant women and HRPs (p=0.012). However, religion did not show a significant association with HRPs (p=.962). Thus, the above factors influence the event of pregnancy (Table 3).

**Table 3:** Association of high-risk pregnancy with demographic variables * p-value significant at <0.05 level ** p-value significant at <0.01 level *** p-value significant at <0.001 level

Variable (n=1889)	Category	High-risk pregnancy F (%)	Normal pregnancy F (%)	Chi-square (Χ²)	p-value
Age	<19	28 (21.4%)	103 (78.6%)	18.072	0.001**
	20-24	210 (26.9%)	572 (73.1%)		
	25-29	190 (26.8%)	519 (73.2%)		
	30-34	91 (38.9%)	143 (61.1%)		
	>35	11 (33.3%)	22 (66.7%)		
Education	Primary school	23 (24.7%)	70 (75.3%)	19.15	0.002**
	Middle school	63 (21.7%)	227 (78.3%)		
	High school	84 (25.5%)	246 (74.5%)		
	Intermediate/diploma	241 (29.2%)	584 (70.8%)		
	Graduate/postgraduate	110 (32.7%)	226 (67.3%)		
	Profession or honour	09 (60.0%)	06 (40.0%)		
Occupation	Housewife	357 (26.8%)	973 (73.2%)	16.35	0.012*
	Unskilled worker	17 (17.9%)	78 (82.1%)		
	Semi-skilled worker	32 (28.1%)	82 (71.9%)		
	Skilled worker	51 (38.3%)	82 (61.7%)		
	Clerical	11 (31.4%)	24 (68.6%)		
	Semi profession	38 (34.9%)	71 (65.1%)		
	Profession	24 (32.9%)	49 (67.1%)		
Income	<3907	01 (50.0%)	01 (50.0%)	17.25	0.004**
	3908-11707	490 (75.5%)	159 (24.5%)		
	11708-19515	542 (73.4%)	196 (26.6%)		
	19516-29199	269 (64.7%)	147 (35.3%)		
	29200-39032	45 (69.2%)	20 (30.8%)		
	39033-78062	12 (63.2%)	07 (36.8%)		
Socioeconomic status	Lower	01 (50.0%)	01 (50.0%)	18.33	0.000***
	Upper lower	994 (74.8%)	335 (25.2%)		
	Lower middle	246 (64.9%)	133 (35.1%)		
	Upper middle	118 (65.9%)	61 (34.1%)		
Religion	Hindu	1134 (72.0%)	440 (28.0%)	.07	0.962
	Muslim	168 (71.2%)	68 (28.8%)		
	Christian	57 (72.2%)	22 (27.8%)		

Among the antenatal mothers, the majority of them were multigravida (52.9%) and were in the second trimester of pregnancy (28.7%). The findings indicate a significant association between primigravida status and HRPs (p=.006), with a higher proportion of primigravida women experiencing HRPs compared to multigravida women. However, trimester of pregnancy and history of abortion did not show significant associations with HRPs (p=0.210 and p=0.110, respectively). These results provide insights into the relationship between obstetrical history variables and the likelihood of experiencing HRPs (Table 4).

**Table 4:** Association of high-risk pregnancies with obstetrical history *p-value significant at <0.01 level

Variable (n=1889)	Category	High-risk pregnancy F (%)	Normal pregnancy F (%)	Chi-square (Χ²)	p-value
Gravida	Primigravida	276 (31.0%)	613 (69.0%)	7.43	0.006*
	Multigravida	254 (25.4%)	746 (74.6%)		
Trimester	Second	389 (28.9%)	958 (71.1%)	1.57	0.210
	Third	141 (26.0%)	401 (74.0%)		
History of Abortion	Yes	467 (28.7%)	1159 (71.3%)	2.55	0.110
	No	63 (24.0%)	200 (76.0%)		

## Discussion

Pregnancy represents a time of great hope and challenge for women, underscoring the importance of early identification and management of HRPs as a crucial step toward preventing maternal mortality and morbidity. This study aimed to ascertain the occurrence of HRPs and their associated factors. Out of 1889 pregnant women attending ANC clinics, 889 (29%) were identified as having HRPs. This aligns with national statistics from the National Health Portal of India, which estimates the prevalence of HRPs in the range of 20% to 30% [3]. Regional studies further illustrate variability, with a community-based study in Haryana reporting an incidence of 31.4% [8], while in Uttar Pradesh, 40.5% of antenatal mothers visiting Community Health Centers (CHCs) were identified as high-risk [9]. Conversely, a record-based retrospective study in Madhya Pradesh found a lower prevalence of HRP at 16.5% [10]. These findings suggest a heterogeneous distribution of HRPs across different states in India. It is imperative to address the variability in HRP prevalence to meet the Sustainable Development Goal (SDG - 3.1), which aims to reduce the maternal mortality rate (MMR) to less than 70 per 10000 population by 2030.

In our study, hypothyroidism emerged as the most prevalent cause (9.6%) of HRPs. This finding resonates with existing literature, which often ranks thyroid disorders as the second most common condition in pregnancy after diabetes mellitus [11]. In the Indian context, hypothyroidism's prevalence typically ranges between 5% and 10%, with Tamil Nadu reporting a magnitude of around 9%, consistent with our findings. A meta-analysis encompassing 61 studies estimated the prevalence of hypothyroidism in Indian pregnant women at approximately 11.07% [12]. Similarly, a study from coastal regions such as Puducherry reported a slightly higher prevalence of hypothyroidism at 11.5%, and an incidence of hyperthyroidism at 1.8% in Puducherry [13]. These findings challenge the misconception that coastal residents may be less susceptible to thyroid disorders. Furthermore, we identified pregnancy-induced hypertension (6.5%) as the second most predominant high-risk condition. Multiple studies conducted across various regions of India have reported incidences of hypertensive disorders of pregnancy (HDP) consistent with our findings, ranging from 4.0% to 12.3% [14]. For instance, a hospital-based study in northeast India revealed 7.3% of pregnancies being affected by HDP [15]. These findings highlight the significance of recognizing regional differences in the occurrence of high-risk conditions to customize ANC and management strategies appropriately.

In our study, severe anemia (3.2%) emerged as the third most common cause of HRPs. Conversely, a study from West Bengal reported a lower prevalence of severe anemia at 0.5%, with a significant proportion of women exhibiting moderate anemia (60.5%) [16]. Additionally, findings from a cross-sectional study in Karnataka reported a notably higher prevalence of severe anemia at 11% [17]. These discrepancies underscore the regional variability in anemia prevalence across India, despite its status as a highly preventable condition. Furthermore, our study revealed that 2.8% of pregnant mothers were affected by Rh incompatibility, consistent with national estimates ranging from 2% to 10% [18]. Rh incompatibility remains a significant concern in pregnancy due to its potential adverse effects on maternal and fetal health which emphasize the necessity of ongoing efforts in prenatal care programs to tackle these conditions, thereby reducing their adverse effects on both maternal and fetal health outcomes.

Additionally, we found that among 1889 pregnant women 2.5% were diagnosed with GDM. This prevalence aligns with broader findings indicating an overall GDM prevalence of 1.3% in India [19]. However, a population-based cohort study in South Delhi reported a notably higher prevalence of GDM, with 19.2% of urban and peri-urban pregnant women affected [20]. Furthermore, our study identified several less common but potentially significant high-risk conditions that can pose risks during pregnancy and childbirth (Table 2). Through our data analysis, we identified several associated factors of HRP, with maternal age demonstrating a statistically significant association. This aligns with extensive evidence from worldwide studies indicating a link between maternal age and HRP, particularly in cases where the age falls below 17 or exceeds 35, or in instances of multiple pregnancies, although the precise etiology remains undefined [21]. Moreover, a prospective study conducted in Ghana reported that teenage pregnant women were particularly prone to anemia [22]. Also, our study revealed significant associations between HRP and factors such as education, occupation, income, and socioeconomic status. These findings are consistent with a longitudinal study from Puducherry, which similarly identified socioeconomic status as a predictor of HRP [23]. Education appears to play a pivotal role, as educated women tend to be more proactive in monitoring their health, diet, medication, and safety during pregnancy. Furthermore, gravidity emerged as another factor associated with HRP, echoing findings from a cross-sectional study in Brazil, which identified multigravida status as a risk factor for HRP [24]. Similarly, a descriptive study in Indonesia highlighted the increased risk associated with multigravida, attributed to the depletion of iron reserves with each childbirth [25].

Pregnancy imposes significant demands on thyroid hormone production, rendering pregnant women more susceptible to hypothyroidism, particularly in cases of genetic diseases or autoimmune disorders. Hormonal replacement therapy stands as the primary treatment for hypothyroidism during pregnancy [9]. Though hypertension in pregnancy remains a leading cause of maternal and neonatal mortality and morbidity worldwide, including India, we identified it as a second major high-risk condition. Iron deficiency anemia disproportionately affects women of reproductive age, with exacerbations during pregnancy leading to adverse fetal and neonatal outcomes [26]. Despite national programs aimed at correcting anemia, its prevalence has remained high over the past four decades, with inadequate nutritional counseling and low compliance with iron-folic acid (IFA) tablets identified as primary hurdles [27]. While Rh incompatibility is irreversible, it poses a significant risk to fetal health [28]. Pregnancy-induced hormonal fluctuations contribute to elevated blood glucose levels, posing risks for both mother and fetus [29]. These findings underscore the urgent need for healthcare providers to proactively address HRP complications through early detection, continuous monitoring, and appropriate treatment. Early health education, effective counseling, active interventions, and medication adherence are essential components of comprehensive care. Awareness and maternal sensitization programs hold the potential to prevent some HRP complications, emphasizing the importance of holistic approaches to maternal healthcare.

While this study offers valuable insights into HRPs, it is important to acknowledge its limitations. Firstly, the study was confined to a single center, potentially limiting the generalizability of its findings to broader populations. Though this study identified the high-risk conditions, we would have also collected the data regarding the existence of these conditions in previous pregnancy/before pregnancy for better interpretation of the findings. Additionally, the outcomes of these pregnancies were not studied, which restricts the ability to assess the impact of high-risk conditions on maternal and fetal health. Despite these limitations, the study's use of a large sample size over two months significantly contributed to the robustness of the findings.

## Conclusions

The hospital-based survey was employed to rule out the occurrence of HRPs and their associated risk factors. Results indicate that approximately one-third of pregnant women were identified as having HRPs, with factors such as age, socioeconomic status, and gravida playing significant roles. Even though the Government of India has initiated the National Health Mission and programs for enhancing maternal and child health, the current study calls for early identification and management of HRPs by healthcare providers. In addition, providing timely interventions reduces MMR and achieves the targets outlined by the SDGs. Looking ahead, it is imperative to implement targeted strategies in the healthcare settings to manage HRPs, enhance access to quality ANC, and ensure that every pregnant woman receives the necessary support and attention to safeguard both maternal and neonatal health.
